# Residents’ breastfeeding knowledge, comfort, practices, and perceptions: results of the Breastfeeding Resident Education Study (BRESt)

**DOI:** 10.1186/s12887-018-1150-7

**Published:** 2018-05-22

**Authors:** Elizabeth Esselmont, Katherine Moreau, Mary Aglipay, Catherine M. Pound

**Affiliations:** 1 University of Ottawa, 451 Smyth Road, Ottawa, ON K1H 8M5 Canada; 2 Children’s Hospital of Eastern Ontario, 401 Smyth Road, Ottawa, ON K1H 8L1 Canada; 3 Children’s Hospital of Eastern Ontario Research Institute, 401 Smyth Road, Ottawa, ON K1H 5B2 Canada

**Keywords:** Assessment, Postgraduate medical education, Residency, Breastfeeding

## Abstract

**Background:**

Physicians have a significant impact on new mothers’ breastfeeding practices. However, physicians’ breastfeeding knowledge is suboptimal. This knowledge deficit could be the result of limited breastfeeding education in residency. This study aimed to explore pediatric residents’ breastfeeding knowledge, comfort level, clinical practices, and perceptions. It also investigated the level and type of education residents receive on breastfeeding and their preferences for improving it.

**Methods:**

Descriptive, cross-sectional, self-reported online questionnaires were sent to all residents enrolled in a Canadian general pediatric residency program, as well as to their program directors. Resident questionnaires explored breastfeeding knowledge, comfort level, clinical practices, perceptions, educational experiences and educational preferences. Program director questionnaires collected data on current breastfeeding education in Canadian centers. For the resident survey, breastfeeding knowledge was calculated as the percent of correct responses. Demographic factors independently associated with overall knowledge score were identified by multiple linear regression. Descriptive statistics were used for the program director survey.

**Results:**

Overall, 201 pediatric residents, and 14 program directors completed our surveys. Residents’ mean overall breastfeeding knowledge score was 71% (95% CI: 69-79%). Only 4% (95% CI: 2-8%) of residents were very comfortable evaluating latch, teaching parents breastfeeding positioning, and addressing parents’ questions regarding breastfeeding difficulties. Over a quarter had not observed a patient breastfeed. Nearly all agreed or strongly agreed that breastfeeding promotion is part of their role. Less than half reported receiving breastfeeding education during residency and almost all wanted more interactive breastfeeding education. According to pediatric program directors, most of the breastfeeding education residents receive is didactic. Less than a quarter of program directors felt that the amount of breastfeeding education provided was adequate.

**Conclusion:**

Pediatric residents in Canada recognize that they play an important role in supporting breastfeeding. Most residents lack the knowledge and training to manage breastfeeding difficulties but are motivated to learn more about breastfeeding. Pediatric program directors recognize the lack of breastfeeding education.

## Background

Infants, mothers, families, and society benefit from breastfeeding [1]. Breastfeeding decreases the incidence of infectious diseases [2, 3], enhances performance on neurocognitive testing [4, 5], decreases the risk of breast [6, 7] and ovarian cancers [6] in mothers, and strengthens the mother-infant bond [8]. Both the American Academy of Pediatrics and the Canadian Pediatric Society advocate for physicians to be knowledgeable about breastfeeding in order to successfully address and manage breastfeeding issues [1, 9]. Yet, multiple studies have shown that physicians’ knowledge of breastfeeding continues to be inadequate and thus, they are unable to counsel mothers appropriately [10–19].

A lack of physician support negatively affects breastfeeding duration [20–23]. As such, there is a need to provide breastfeeding education to residents. A recent study showed that Canadian physicians’ breastfeeding knowledge is suboptimal, and that breastfeeding education in residency is limited [18]. Although this study provided useful information about specific knowledge gaps among Canadian physicians, it included only a small number of pediatric residents. Therefore, we undertook the present Canadian study to explore: (a) residents’ breastfeeding knowledge, comfort level, clinical practices, and perceptions; (b) the level and type of education residents receive on breastfeeding and their preferences for improving it; and (c) whether demographic variables are associated with residents’ breastfeeding knowledge, comfort level, clinical practices, and perceptions. We also surveyed pediatric program directors in Canada to gather additional information on the level of breastfeeding education in their programs.

## Methods

### Study design and participant criteria

We conducted a descriptive, cross-sectional, self-reported, online survey of all general pediatric residents enrolled in years 1 to 4 of a Canadian residency training program in March 2014. There were 638 general pediatric residents enrolled in Canadian programs for the 2013-2014 academic year. Residents were excluded if they had already entered a pediatric subspecialty training program or if they had previously been enrolled in a residency training program outside of Canada. We also conducted a descriptive, cross-sectional, self-reported, online survey of the 17 pediatric residency program directors across Canada.

### Sample size

We based our sample size calculation on the response rate (30%) of a previous national breastfeeding study as well as other studies involving physicians [18, 24]. Given that 637 residents were eligible for the study, a response rate of 30% would equate to 191 participants. This would allow us to estimate a proportion (assuming a true value of 50%) to within plus or minus 7.1% with 95% confidence. We expected all 17 Canadian program directors to participate.

### Survey instruments

#### Resident survey

The resident survey included the following six domains: breastfeeding knowledge, comfort level, clinical practices, perceptions, educational experiences, and educational preferences. To develop questions for the knowledge domain, we drew on items from the American Academy of Pediatrics’ online breastfeeding curriculum pre- and post- knowledge tests [25, 26] as well those from a previous physician survey on breastfeeding [18]. We obtained permission from both these sources prior to using their items. We also ensured that all survey questions reflected the National Board of Medical Examiners guidelines for valid one-best answer knowledge questions [27]. Five International Board-Certified Lactation consultants and 12 subspecialty pediatric residents reviewed the survey for clarity and feasibility. We then modified the survey based on the feedback received from these individuals.

The final survey consisted of 35 closed-ended questions. These questions included ten multiple-choice items for the knowledge domain. We defined a priori an overall knowledge score of 70% (7/10 questions correct) as acceptable because this cut-off score was used in the previous national physician breastfeeding survey [18]. The survey also included three questions for each of the comfort level, clinical practices, perceptions, educational experiences, and educational preferences domains. The remaining questions addressed participant eligibility and demographics.

#### Program director survey

A three-question program director survey was also developed by the study team to gather data on current breastfeeding education in Canadian pediatric residency programs. The following questions were included: (1) In total, how much time does your program devote to educating residents about the assessment and management of breastfeeding difficulties? (2) What modalities are used to educate residents in your program about breastfeeding? (3) To what extent do you agree that the amount of breastfeeding education currently provided to residents in your program is adequate? Given the exploratory nature of these questions and the brevity of the survey, validity evidence was not established for this survey.

### Data collection

The Children’s Hospital of Eastern Ontario Research Ethics Board approved this study. All pediatric program directors consented for residents in their programs to participate. Each program director provided the study team with the contact information of one resident representative, who distributed the online resident survey. This survey was available in Canada’s two official languages (French and English). The online program director survey was sent directly to all program directors via e-mail.

### Statistical analysis

For the resident survey, descriptive statistics were used to summarize the respondents’ demographic characteristics, educational experiences, comfort level, clinical practice, perceptions, and educational preferences. Breastfeeding knowledge was calculated as the percent of correct responses. Multiple linear regression was used to identify demographic factors that were independently associated with the overall knowledge score. All demographic variables were included in the multivariate model. Missing data were handled via list-wise deletion. The identification of demographic factors associated with knowledge was the primary goal of the modeling so only main effects (no interactions) were assessed. Associations between residents’ comfort level and these demographic factors were tested using chi-squared tests and Fisher’s exact test where appropriate. A *p*-value of < 0.05 was considered statistically significant. The analyses were performed using SPSS (IBM Corp. Version 21.0.). Descriptive statistics were also used to summarize time spent on breastfeeding education in Canadian pediatric residency programs, levels of education provided and program directors perceptions regarding the adequacy of breastfeeding teaching provided to their residents.

For the program director survey, descriptive statistics were used to summarize time spent on breastfeeding education in Canadian pediatric residency programs, levels of education provided, and program directors’ perceptions regarding the adequacy of the breastfeeding education.

## Results

### Resident survey

#### Respondent characteristics

The majority of respondents were female (88%) and under the age of 30 (70%). Of the respondents, 18% of had children. Table 1 provides additional characteristics of the respondents.

**Table 1:** Participant Demographics

Characteristic	Total *n* (%)
Age (years) ^a^	
< 30	140 (70)
30-50	60 (30)
Current Year of Residency Training	
First	57 (28)
Second	53 (26)
Third	57 (28)
Fourth	34 (17)
Province / Territory of Residency Program^b^	
Western Provinces (BC, AB, SK, MB)^c^	49 (25)
Ontario	75 (38)
Quebec	57 (29)
Atlantic Provinces (NS, NFLD)^d^	19 (10)
Breastfeeding learning (check all that apply)	
Personal experience	54 (27)
Medical school	78 (39)
Residency	134 (67)
Course	9 (5)
Self-directed learning	70 (35)
Other	17 (9)
Breastfeeding education during residency	97 (48)
Certification in breastfeeding support	1 (0.5)
Has one or more children	36 (18)

^a^Missing 1 response

^b^Missing 1 response

^c^BC: British Columbia, AB: Alberta, SK: Saskatchewan, MB: Manitoba

^d^NS: Nova Scotia, NFLD: Newfoundland

### Knowledge

The average overall knowledge score of pediatric residents was 71% (95%CI: 69-73%). Table 2 summarizes residents’ results on each question in the knowledge domain of the questionnaire. Multiple linear regression results are shown in Table 3. Residents who had one or more children and residents who reported receiving breastfeeding education scored an average of 11.6 points higher (95% CI: 3.1-22.7; *p* = 0.01) and an average of 4.8 points higher (95% CI: -0.07-9.7; *p* = 0.05) (on a 100-point scale), respectively, on the knowledge domain of the questionnaire.

**Table 2:** Frequency Distribution of Correct Response on Residents’ Survey (*N* = 201)

Question	Correct Answer	Respondents with Correct Answer (*n* %)
A mother complains that her otherwise healthy and thriving 6 week old infant has been breastfeeding almost every 1-2 h for a day or two. What do you explain to her?	The baby requires more milk because he/she is growing and frequent breastfeeding is his/her way to increase milk supply.	145 (72.9)^a^
A mother with a 3-day-old baby presents with sore nipples. The problem began with the first feeding and has persisted with every feeding. What is the most likely source of the problem? (AAP)^b^	Poor attachment to the breast	175 (87.1)
Which of the following is a correct statement about the latch during breastfeeding? (AAP)	The baby needs to be latched so that he compresses the milk sinuses when suckling at the breast^e^	52 (25.9)
The mother of a breastfed 3-month-old will be away from her baby overnight for a business trip. She has an electric pump, but will not have a refrigerator available to her during the trip. Of the following, which is the BEST advice to give her regarding pumping and storing of her breast milk during the time of separation? (AAP)	She should pump the milk and store it with ice in a cooler at approximately refrigerator temperature (< 4 °C)	102 (50.7)
What increases milk production? (AAP)	More frequent milk removal	175 (87.5)^c^
During the postpartum stay, a breastfeeding mother reports that she is having difficulty getting her infant to breastfeed. What is the best way to manage this situation? (AAP)	Request assistance for the mother at the infant’s next feeding to evaluate the breastfeeding technique	192 (96.0)^d^
What is severe engorgement most often due to? (AAP)	Infrequent feedings	140 (69.7)
An otherwise healthy, well-hydrated 5-day old breastfeeding infant is admitted to the hospital with jaundice. In addition to treating the child with phototherapy, which of the following would you first recommend?	Feed the infant more frequently and ensure the mother knows how and when to use a breast pump	153 (76.1)
In a baby who is breastfeeding effectively, what is the position of the baby’s tongue?	The tongue is easily visible	102 (50.7)
What is the first thing you would do if a breastfeeding mother complains that her nipples are cracked and sore?	Assess baby’s position and latch	192 (95.5)

^a^Missing 2 responses

^b^AAP: American Academy of Pediatrics

^c^Missing 1 response

^d^Missing 1 response

^e^This answer, taken verbatim from the American Academy of Pediatrics, is only partially correct as milk sinuses have been demonstrated to be absent from the breast as per Ramsay et al. study [29]

**Table 3:** Unadjusted and Adjusted^a^ Linear Regression for Breastfeeding Knowledge Score among Canadian Pediatric Residents (*n* = 191)

	Unadjusted		Adjusted	
	Estimate (95% CI)	*P* Value	Estimate (95% CI)	*P* Value
Age		0.28		0.45
< 30	Reference		Reference	
30-50	2.7 (− 2.2-7.6)		−2.23 (− 8.0-3.5)	
Gender		0.05		0.08
Male	Reference		Reference	
Female	6.9 (0.02-13.7)		6.5 (−0.8-13.8)	
Current Year of Residency Training		0.01		0.08
First	Reference		Reference	
Second	0.2 (−5.7-6.1)		−0.8 (−7.1-5.5)	
Third	6.1 (0.3-11.9)		4.4 (−1.8-10.7)	
Fourth	10.1 (3.4-16.8)		7.9 (0.04-15.8)	
Province / Territory of Residency Program		0.06		0.13
Western Provinces (BC, AB, SK, MB)^b^	Reference		Reference	
Ontario	−1.7 (−7.4-4.1)		−0.2 (−5.9-5.5)	
Quebec	2.1 (−4.0-8.2)		3.2 (−3.2-9.6)	
Atlantic Provinces	9.2 (0.8-17.8)		8.8 (0.2-17.4)	
Breastfeeding learning				
Personal experience	4.9 (−0.1-9.9)	0.06	−1.5 (−9.6-6.4)	0.70
Medical school	0.6 (−4.0-5.2)	0.80	4.4 (−0.5-9.4)	0.08
Residency	1.6 (−3.2-6.3)	0.52	−1.4 (−6.7-3.9)	0.60
Course	2.4 (−8.4-13.2)	0.66	−11.3 (−23.2-0.6)	0.06
Self-directed learning	2.6 (−2.1-7.3)	0.28	1.9 (−2.7-6.5)	0.43
Other	−1.8 (−9.8-6.3)	0.66	−0.1 (−8.4-8.2)	0.98
Breastfeeding Education During Residency	6.1 (1.7-10.5)	0.01	4.8 (−0.07-9.7)	0.05
Certification in breastfeeding support	19.5 (−12.7-50.8)	0.24	8.1 (−24.7-41.0)	0.63
Has one or more children	11.6 (6.0-17.2)	< 0.01	12.9 (3.1-22.7)	0.01

^a^Adjusted regression model includes all variables in the table

^b^BC: British Columbia, AB: Alberta, SK: Saskatchewan, MB: Manitoba

### Comfort level

Seven percent of residents were very comfortable evaluating an infant’s latch at the breast while 10% were very comfortable teaching a parent to position an infant at the breast. Nine percent were very comfortable addressing parents’ questions about breastfeeding difficulties and 4 % of residents were very comfortable at all three of these skills. Older age (30-50 years old versus less than 30), higher year of residency training, personal experience with breastfeeding, having one or more children, and possessing certification in breastfeeding were all associated with higher levels of comfort with these skills (*p* < 0.05).

### Clinical practices supporting breastfeeding

A total of 28% of residents had not observed a patient breastfeed in the hospital or office setting; 74% had observed a patient breastfeed 4 times or less in the same setting. In addition, 75 and 96% of residents reported having never taught a new mother breastfeeding techniques (e.g., latch, positioning at the breast), and how to use a breast pump, respectively.

### Perceptions

Ninety-four percent of respondents agreed or strongly agreed that breastfeeding promotion is part of their role as a pediatric resident; 54% agreed or strongly agreed that is the responsibility of the child’s primary physician to perform an evaluation of breastfeeding in the first 3 to 5 days after birth, and 92% of residents agreed or strongly agreed that physicians can influence how long a mother decides to breastfeed.

### Quality of education

Less than half (48%) of pediatric residents reported receiving breastfeeding education during their residency. The teaching provided was most often didactic. Only 16% of pediatric residents had the opportunity to follow a lactation consultant during their residency. Only 5% of pediatric residents reported receiving teaching in the form of interactive workshops with breastfeeding mothers.

### Educational preferences

Almost all residents (93%) agreed or strongly agreed that more breastfeeding education should be incorporated into pediatric residency training. Most residents felt that 4-8 h of breastfeeding teaching throughout residency would be the optimal amount. The two teaching methods that residents believed would be most effective for helping them learn about breastfeeding were following a lactation consultant (81%) and interactive workshops with breastfeeding mothers (71%).

### Program directors’ survey

Information on 14 residency programs was obtained from the program directors, for a response rate of 82%. The results of the Program Directors’ Survey are summarized in Table 4. A total of 21% of program directors surveyed felt that the amount of breastfeeding education provided by their program was adequate.

**Table 4:** Program Director Survey (*N* = 14)

Question	Answer	Respondents *n* (%)
How much time does your program devote to educating residents about assessment and management of breastfeeding difficulties (total over 4 years of residency)?	0 h	1 (7.1)
	1-3 h	4 (28.6)
	4-8 h	7 (50.0)
	9-16 h	2 (14.3)
	more than 16 h	0 (0.0)
What modalities are used to educate residents in your program about breastfeeding? (pick all that apply)	None	1 (7.1)
	Didactic teaching	9 (64.3)
	Grand rounds	2 (14.3)
	Computer based tutorials	3 (21.4)
	Interactive workshops with moms	1 (7.1)
	Following lactation consultant	4 (28.6)
	Other	4 (28.6)
To what extent do you agree that the amount of breastfeeding education currently provided to residents in your program is adequate?	Strongly Disagree	1 (7.1)
	Disagree	6 (42.9)
	Neutral	4 (28.6)
	Agree	3 (21.4)

## Discussion

Although residents scored slightly above our minimum acceptable score of 70% on the knowledge domain of the survey, there is still significant room for improving pediatric residents’ breastfeeding knowledge. Residents’ comfort level with assessing and managing breastfeeding difficulties was suboptimal. Although most residents agree that it is their role to promote and assist with breastfeeding, many have never watched a patient being breastfeed. Our study showed that residents receive very little breastfeeding education. Nearly all residents felt that more breastfeeding education should be incorporated into their residency. This feeling is shared by most program directors, who identified a need for more breastfeeding education in residency.

Our survey identified some specific knowledge gaps among pediatric residents, particularly in the area of latch assessment. Other poorly answered questions related to the pumping and storage of breastmilk, as well as an infant’s tongue position while feeding. This suggests that pediatric residents lack knowledge about some of the practical aspects of breastfeeding. These results are in line with those of other studies worldwide [10, 17–19]. In Canada, a recent study also showed that failing to identify aspects of a successful latch was a significant knowledge gap for family physicians and pediatricians [18].

Given the above results, it is not surprising that very few residents felt comfortable providing parents with assistance in the practical aspects of breastfeeding. Only 8 of our 201 participants felt very comfortable evaluating an infant’s latch at the breast, teaching a parent how to position an infant at the breast, and addressing parents’ questions about breastfeeding issues. This result is quite different from the results of a recent Canadian study [18], where 75% of physicians in the study reported feeling comfortable addressing breastfeeding difficulties. In that previous study, this number dropped significantly when physicians were asked specifically about their comfort with technical breastfeeding skills, such as latch assessment, milk transfer and teaching mothers how to use a breast pump. This previous study suggested that perhaps physicians overestimated their overall comfort with breastfeeding as they may not be aware of their deficiencies. The residents in our current study reported significantly lower comfort levels with addressing breastfeeding difficulties, indicating that residents may be more aware of their gaps in breastfeeding knowledge and skills, and may therefore be more receptive to educational interventions.

Not surprisingly, having children, personal experience with breastfeeding, and certification in breastfeeding were all associated with greater comfort level with the practical aspects of breastfeeding and counseling. Interestingly, higher year of residency training was also associated with higher self-reported comfort. However, this increase in comfort was not paralleled by an increase in knowledge, which raises the possibility that residents may feel more confident without necessarily being more proficient.

We found that residents had very limited experience observing their patients being breastfed. This may be due to residents’ awareness of their lack of breastfeeding counseling-related knowledge, resulting in residents feeling ill-equipped to help if breastfeeding difficulties arise and therefore avoiding these vulnerable situations. This is particularly concerning since previous studies have established that a lack of physician support negatively impacts breastfeeding duration [20–23]. Nevertheless, we were encouraged to find that despite their discomfort with assessing and managing some of the practical aspects of breastfeeding, residents have positive perceptions about breastfeeding. Of the pediatric residents who completed our survey, the vast majority agreed that breastfeeding promotion is part of their role as a pediatric resident.

Our results clearly demonstrate that breastfeeding education for pediatric residents in Canada could be greatly improved. The small number of residents who had received breastfeeding education reported that the teaching received was mostly didactic, which corresponds to what the program directors reported. However, when residents were asked about their preferred mode of breastfeeding education, most felt that interactive workshops with breastfeeding mothers or shadowing a lactation consultant would be the best ways to learn. Evidently there is a gap between the breastfeeding education residents are currently receiving and the type of breastfeeding education they perceive would be most beneficial. This shows a significant area for improvement within pediatric residency programs across Canada.

Study limitations include a somewhat low overall response rate, although we expected this for the population surveyed [24]. Given that residents self-selected to complete the survey, this could have resulted in a higher proportion of residents with a breastfeeding interest participating in our study. Such self-selection would be expected to positively skew our results, suggesting that the overall knowledge score of all pediatric residents is actually below the 71% score that we obtained. We specifically designed the survey used in this study and the knowledge items have not been widely used or tested from a psychometric point of view. However, it is important to note that we adapted questions from other studies used to assess physicians’ breastfeeding knowledge, confidence, beliefs, and attitudes and as such, we were able to make comparisons between our results and those of the other studies.

This is the largest survey of pediatric residents in Canada assessing breastfeeding knowledge. Moreover, we obtained information from the majority of program directors, giving us a complete view of the state of breastfeeding education among pediatric residents in Canada.

## Conclusion

Though the Canadian Pediatric Society, the American Academy of Pediatrics and the World Health Organization all emphasize the important role physicians play in the initiation and continuation of breastfeeding [1, 9, 28], the results of our study show that pediatric residents across Canada are inadequately prepared for this role during their residency. Based on these results, we suggest that a breastfeeding curriculum be implemented in Canadian pediatric residency training programs to ensure that residents are prepared to assess and address breastfeeding difficulties that arise in their patients. We hope that by addressing this education deficit during residency, we can train pediatricians who can provide quality care to breastfeeding infants and mothers and thereby increase the rates and duration of breastfeeding.
